# Supplementary material for: Resolving the full spectrum of human genome variation using Linked-Reads
Source: Genome Res. 2019 Apr;29(4):635–45. doi: 10.1101/gr.234443.118 (PMC6442396; doi:10.1101/gr.234443.118)
Supplement: Supplemental Material [file supp_gr.234443.118_Supplemental_longranger-2.2.zip › longranger-2.2.tar.bz2!/lr2.2-public/lib/stan/cmdstan/stan/doc/api/html/agrad_2rev_2matrix_2mdivide__left__spd_8hpp_source.html]

Stan: src/stan/agrad/rev/matrix/mdivide\_left\_spd.hpp Source File


|  |  |
| --- | --- |
|  | Stan  2.5.0  probability, sampling & optimization |


- Main Page
- Related Pages
- Namespaces
- Classes
- Files

- File List
- File Members

All Classes Namespaces Files Functions Variables Typedefs Enumerations Enumerator Friends Macros Pages

- src
- stan
- agrad
- rev
- matrix

mdivide\_left\_spd.hpp

Go to the documentation of this file.

1 #ifndef STAN\_\_AGRAD\_\_REV\_\_MATRIX\_\_MDIVIDE\_LEFT\_SPD\_HPP

2 #define STAN\_\_AGRAD\_\_REV\_\_MATRIX\_\_MDIVIDE\_LEFT\_SPD\_HPP

3

4 #include <vector>

5 #include <stan/math/matrix/Eigen.hpp>

6 #include <stan/math/matrix/typedefs.hpp>

7 #include <stan/math/error\_handling/matrix/check\_multiplicable.hpp>

8 #include <stan/agrad/rev/var.hpp>

9 #include <stan/agrad/rev/matrix/typedefs.hpp>

10 #include <stan/math/error\_handling/matrix/check\_square.hpp>

11

12 namespace stan {

13  namespace agrad {

14

15  namespace {

16  template <int R1,int C1,int R2,int C2>

17  class mdivide\_left\_spd\_alloc : public chainable\_alloc {

18  public:

19  virtual ~mdivide\_left\_spd\_alloc() {}

20

21  Eigen::LLT< Eigen::Matrix<double,R1,C1> > \_llt;

22  Eigen::Matrix<double,R2,C2> C\_;

23  };

24

25  template <int R1,int C1,int R2,int C2>

26  class mdivide\_left\_spd\_vv\_vari : public vari {

27  public:

28  int M\_; // A.rows() = A.cols() = B.rows()

29  int N\_; // B.cols()

30  vari\*\* \_variRefA;

31  vari\*\* \_variRefB;

32  vari\*\* \_variRefC;

33  mdivide\_left\_spd\_alloc<R1,C1,R2,C2> \*\_alloc;

34

35  mdivide\_left\_spd\_vv\_vari(const Eigen::Matrix<var,R1,C1> &A,

36  const Eigen::Matrix<var,R2,C2> &B)

37  : vari(0.0),

38  M\_(A.rows()),

39  N\_(B.cols()),

40  \_variRefA((vari\*\*)stan::agrad::memalloc\_.alloc(sizeof(vari\*)

41  \* A.rows() \* A.cols())),

42  \_variRefB((vari\*\*)stan::agrad::memalloc\_.alloc(sizeof(vari\*)

43  \* B.rows() \* B.cols())),

44  \_variRefC((vari\*\*)stan::agrad::memalloc\_.alloc(sizeof(vari\*)

45  \* B.rows() \* B.cols())),

46  \_alloc(new mdivide\_left\_spd\_alloc<R1,C1,R2,C2>())

47  {

48  using Eigen::Matrix;

49  using Eigen::Map;

50

51  Matrix<double,R1,C1> Ad(A.rows(),A.cols());

52

53  size\_t pos = 0;

54  for (size\_type j = 0; j < M\_; j++) {

55  for (size\_type i = 0; i < M\_; i++) {

56  \_variRefA[pos] = A(i,j).vi\_;

57  Ad(i,j) = A(i,j).val();

58  pos++;

59  }

60  }

61

62  pos = 0;

63  \_alloc->C\_.resize(M\_,N\_);

64  for (size\_type j = 0; j < N\_; j++) {

65  for (size\_type i = 0; i < M\_; i++) {

66  \_variRefB[pos] = B(i,j).vi\_;

67  \_alloc->C\_(i,j) = B(i,j).val();

68  pos++;

69  }

70  }

71

72  \_alloc->\_llt = Ad.llt();

73  \_alloc->\_llt.solveInPlace(\_alloc->C\_);

74

75  pos = 0;

76  for (size\_type j = 0; j < N\_; j++) {

77  for (size\_type i = 0; i < M\_; i++) {

78  \_variRefC[pos] = new vari(\_alloc->C\_(i,j),false);

79  pos++;

80  }

81  }

82  }

83

84  virtual void chain() {

85  using Eigen::Matrix;

86  using Eigen::Map;

87  Eigen::Matrix<double,R1,C1> adjA(M\_,M\_);

88  Eigen::Matrix<double,R2,C2> adjB(M\_,N\_);

89

90  size\_t pos = 0;

91  for (size\_type j = 0; j < N\_; j++)

92  for (size\_type i = 0; i < M\_; i++)

93  adjB(i,j) = \_variRefC[pos++]->adj\_;

94

95  \_alloc->\_llt.solveInPlace(adjB);

96  adjA.noalias() = -adjB \* \_alloc->C\_.transpose();

97

98  pos = 0;

99  for (size\_type j = 0; j < M\_; j++)

100  for (size\_type i = 0; i < M\_; i++)

101  \_variRefA[pos++]->adj\_ += adjA(i,j);

102

103  pos = 0;

104  for (size\_type j = 0; j < N\_; j++)

105  for (size\_type i = 0; i < M\_; i++)

106  \_variRefB[pos++]->adj\_ += adjB(i,j);

107  }

108  };

109

110  template <int R1,int C1,int R2,int C2>

111  class mdivide\_left\_spd\_dv\_vari : public vari {

112  public:

113  int M\_; // A.rows() = A.cols() = B.rows()

114  int N\_; // B.cols()

115  vari\*\* \_variRefB;

116  vari\*\* \_variRefC;

117  mdivide\_left\_spd\_alloc<R1,C1,R2,C2> \*\_alloc;

118

119  mdivide\_left\_spd\_dv\_vari(const Eigen::Matrix<double,R1,C1> &A,

120  const Eigen::Matrix<var,R2,C2> &B)

121  : vari(0.0),

122  M\_(A.rows()),

123  N\_(B.cols()),

124  \_variRefB((vari\*\*)stan::agrad::memalloc\_.alloc(sizeof(vari\*)

125  \* B.rows() \* B.cols())),

126  \_variRefC((vari\*\*)stan::agrad::memalloc\_.alloc(sizeof(vari\*)

127  \* B.rows() \* B.cols())),

128  \_alloc(new mdivide\_left\_spd\_alloc<R1,C1,R2,C2>())

129  {

130  using Eigen::Matrix;

131  using Eigen::Map;

132

133  size\_t pos = 0;

134  \_alloc->C\_.resize(M\_,N\_);

135  for (size\_type j = 0; j < N\_; j++) {

136  for (size\_type i = 0; i < M\_; i++) {

137  \_variRefB[pos] = B(i,j).vi\_;

138  \_alloc->C\_(i,j) = B(i,j).val();

139  pos++;

140  }

141  }

142

143  \_alloc->\_llt = A.llt();

144  \_alloc->\_llt.solveInPlace(\_alloc->C\_);

145

146  pos = 0;

147  for (size\_type j = 0; j < N\_; j++) {

148  for (size\_type i = 0; i < M\_; i++) {

149  \_variRefC[pos] = new vari(\_alloc->C\_(i,j),false);

150  pos++;

151  }

152  }

153  }

154

155  virtual void chain() {

156  using Eigen::Matrix;

157  using Eigen::Map;

158  Eigen::Matrix<double,R2,C2> adjB(M\_,N\_);

159

160  size\_t pos = 0;

161  for (size\_type j = 0; j < adjB.cols(); j++)

162  for (size\_type i = 0; i < adjB.rows(); i++)

163  adjB(i,j) = \_variRefC[pos++]->adj\_;

164

165  \_alloc->\_llt.solveInPlace(adjB);

166

167  pos = 0;

168  for (size\_type j = 0; j < adjB.cols(); j++)

169  for (size\_type i = 0; i < adjB.rows(); i++)

170  \_variRefB[pos++]->adj\_ += adjB(i,j);

171  }

172  };

173

174  template <int R1,int C1,int R2,int C2>

175  class mdivide\_left\_spd\_vd\_vari : public vari {

176  public:

177  int M\_; // A.rows() = A.cols() = B.rows()

178  int N\_; // B.cols()

179  vari\*\* \_variRefA;

180  vari\*\* \_variRefC;

181  mdivide\_left\_spd\_alloc<R1,C1,R2,C2> \*\_alloc;

182

183  mdivide\_left\_spd\_vd\_vari(const Eigen::Matrix<var,R1,C1> &A,

184  const Eigen::Matrix<double,R2,C2> &B)

185  : vari(0.0),

186  M\_(A.rows()),

187  N\_(B.cols()),

188  \_variRefA((vari\*\*)stan::agrad::memalloc\_.alloc(sizeof(vari\*)

189  \* A.rows() \* A.cols())),

190  \_variRefC((vari\*\*)stan::agrad::memalloc\_.alloc(sizeof(vari\*)

191  \* B.rows() \* B.cols())),

192  \_alloc(new mdivide\_left\_spd\_alloc<R1,C1,R2,C2>())

193  {

194  using Eigen::Matrix;

195  using Eigen::Map;

196

197  Matrix<double,R1,C1> Ad(A.rows(),A.cols());

198

199  size\_t pos = 0;

200  for (size\_type j = 0; j < M\_; j++) {

201  for (size\_type i = 0; i < M\_; i++) {

202  \_variRefA[pos] = A(i,j).vi\_;

203  Ad(i,j) = A(i,j).val();

204  pos++;

205  }

206  }

207

208  \_alloc->\_llt = Ad.llt();

209  \_alloc->C\_ = \_alloc->\_llt.solve(B);

210

211  pos = 0;

212  for (size\_type j = 0; j < N\_; j++) {

213  for (size\_type i = 0; i < M\_; i++) {

214  \_variRefC[pos] = new vari(\_alloc->C\_(i,j),false);

215  pos++;

216  }

217  }

218  }

219

220  virtual void chain() {

221  using Eigen::Matrix;

222  using Eigen::Map;

223  Eigen::Matrix<double,R1,C1> adjA(M\_,M\_);

224  Eigen::Matrix<double,R1,C2> adjC(M\_,N\_);

225

226  size\_t pos = 0;

227  for (size\_type j = 0; j < adjC.cols(); j++)

228  for (size\_type i = 0; i < adjC.rows(); i++)

229  adjC(i,j) = \_variRefC[pos++]->adj\_;

230

231  adjA = -\_alloc->\_llt.solve(adjC\*\_alloc->C\_.transpose());

232

233  pos = 0;

234  for (size\_type j = 0; j < adjA.cols(); j++)

235  for (size\_type i = 0; i < adjA.rows(); i++)

236  \_variRefA[pos++]->adj\_ += adjA(i,j);

237  }

238  };

239  }

240

241  template <int R1,int C1,int R2,int C2>

242  inline

243  Eigen::Matrix<var,R1,C2>

244  mdivide\_left\_spd(const Eigen::Matrix<var,R1,C1> &A,

245  const Eigen::Matrix<var,R2,C2> &b) {

246  Eigen::Matrix<var,R1,C2> res(b.rows(),b.cols());

247

248  stan::math::check\_square("mdivide\_left\_spd(%1%)",A,"A",(double\*)0);

249  stan::math::check\_multiplicable("mdivide\_left\_spd(%1%)",A,"A",

250  b,"b",(double\*)0);

251

252  // NOTE: this is not a memory leak, this vari is used in the

253  // expression graph to evaluate the adjoint, but is not needed

254  // for the returned matrix. Memory will be cleaned up with the arena allocator.

255  mdivide\_left\_spd\_vv\_vari<R1,C1,R2,C2> \*baseVari = new mdivide\_left\_spd\_vv\_vari<R1,C1,R2,C2>(A,b);

256

257  size\_t pos = 0;

258  for (size\_type j = 0; j < res.cols(); j++)

259  for (size\_type i = 0; i < res.rows(); i++)

260  res(i,j).vi\_ = baseVari->\_variRefC[pos++];

261

262  return res;

263  }

264

265  template <int R1,int C1,int R2,int C2>

266  inline

267  Eigen::Matrix<var,R1,C2>

268  mdivide\_left\_spd(const Eigen::Matrix<var,R1,C1> &A,

269  const Eigen::Matrix<double,R2,C2> &b) {

270  Eigen::Matrix<var,R1,C2> res(b.rows(),b.cols());

271

272  stan::math::check\_square("mdivide\_left\_spd(%1%)",A,"A",(double\*)0);

273  stan::math::check\_multiplicable("mdivide\_left\_spd(%1%)",A,"A",

274  b,"b",(double\*)0);

275

276  // NOTE: this is not a memory leak, this vari is used in the

277  // expression graph to evaluate the adjoint, but is not needed

278  // for the returned matrix. Memory will be cleaned up with the arena allocator.

279  mdivide\_left\_spd\_vd\_vari<R1,C1,R2,C2> \*baseVari = new mdivide\_left\_spd\_vd\_vari<R1,C1,R2,C2>(A,b);

280

281  size\_t pos = 0;

282  for (size\_type j = 0; j < res.cols(); j++)

283  for (size\_type i = 0; i < res.rows(); i++)

284  res(i,j).vi\_ = baseVari->\_variRefC[pos++];

285

286  return res;

287  }

288

289  template <int R1,int C1,int R2,int C2>

290  inline

291  Eigen::Matrix<var,R1,C2>

292  mdivide\_left\_spd(const Eigen::Matrix<double,R1,C1> &A,

293  const Eigen::Matrix<var,R2,C2> &b) {

294  Eigen::Matrix<var,R1,C2> res(b.rows(),b.cols());

295

296  stan::math::check\_square("mdivide\_left\_spd(%1%)",A,"A",(double\*)0);

297  stan::math::check\_multiplicable("mdivide\_left\_spd(%1%)",A,"A",

298  b,"b",(double\*)0);

299

300  // NOTE: this is not a memory leak, this vari is used in the

301  // expression graph to evaluate the adjoint, but is not needed

302  // for the returned matrix. Memory will be cleaned up with the arena allocator.

303  mdivide\_left\_spd\_dv\_vari<R1,C1,R2,C2> \*baseVari = new mdivide\_left\_spd\_dv\_vari<R1,C1,R2,C2>(A,b);

304

305  size\_t pos = 0;

306  for (size\_type j = 0; j < res.cols(); j++)

307  for (size\_type i = 0; i < res.rows(); i++)

308  res(i,j).vi\_ = baseVari->\_variRefC[pos++];

309

310  return res;

311  }

312

313  }

314 }

315 #endif

check\_multiplicable.hpp

\_variRefB

vari \*\* \_variRefB

**Definition:** mdivide\_left\_spd.hpp:31

typedefs.hpp

typedefs.hpp

stan::agrad::memalloc\_

memory::stack\_alloc memalloc\_

**Definition:** var\_stack.cpp:16

N\_

int N\_

**Definition:** mdivide\_left\_spd.hpp:29

\_variRefC

vari \*\* \_variRefC

**Definition:** mdivide\_left\_spd.hpp:32

stan::math::check\_multiplicable

bool check\_multiplicable(const char \*function, const T1 &y1, const char \*name1, const T2 &y2, const char \*name2, T\_result \*result)

**Definition:** check\_multiplicable.hpp:15

stan::agrad::size\_type

Eigen::Matrix< double, Eigen::Dynamic, Eigen::Dynamic >::Index size\_type

**Definition:** typedefs.hpp:14

stan::math::rows

size\_t rows(const Eigen::Matrix< T, R, C > &m)

**Definition:** rows.hpp:12

\_llt

Eigen::LLT< Eigen::Matrix< double, R1, C1 > > \_llt

**Definition:** mdivide\_left\_spd.hpp:21

\_variRefA

vari \*\* \_variRefA

**Definition:** mdivide\_left\_spd.hpp:30

var.hpp

stan::math::cols

size\_t cols(const Eigen::Matrix< T, R, C > &m)

**Definition:** cols.hpp:12

Eigen.hpp

M\_

int M\_

**Definition:** mdivide\_left\_spd.hpp:28

\_alloc

mdivide\_left\_spd\_alloc< R1, C1, R2, C2 > \* \_alloc

**Definition:** mdivide\_left\_spd.hpp:33

stan::agrad::mdivide\_left\_spd

Eigen::Matrix< var, R1, C2 > mdivide\_left\_spd(const Eigen::Matrix< var, R1, C1 > &A, const Eigen::Matrix< var, R2, C2 > &b)

**Definition:** mdivide\_left\_spd.hpp:244

C\_

Eigen::Matrix< double, R2, C2 > C\_

**Definition:** mdivide\_left\_spd.hpp:22

stan::math::check\_square

bool check\_square(const char \*function, const Eigen::Matrix< T\_y, Eigen::Dynamic, Eigen::Dynamic > &y, const char \*name, T\_result \*result)

Return true if the specified matrix is square.

**Definition:** check\_square.hpp:24

check\_square.hpp


---

[ Stan Home Page ]

*© 2011–2014,
Stan Development Team.*
